# Cutaneous Necrosis Over the Nose and Lower Limbs Induced by Acenocoumarol: A Case Report and Literature Review

**DOI:** 10.7759/cureus.36960

**Published:** 2023-03-31

**Authors:** Maha M Alsahli, Asem Shadid, Arwa Al-Modayfer, Frederic Cambazard, Jean-Luc Perrot

**Affiliations:** 1 Dermatology, King Saud Medical City, Riyadh, SAU; 2 Dermatology, King Fahad Medical City, Riyadh, SAU; 3 Dermatology, University Hospital of Saint-Étienne, Saint-Étienne, FRA

**Keywords:** protein c, leukocytoclastic vasculitis, acenocoumarol, oral anticoagulant therapy, skin necrosis

## Abstract

Coumarin derivatives are the most used class of oral anticoagulants, and almost 1-2% of adults worldwide take it in the form of warfarin (WA) or acenocoumarol (AC). Cutaneous necrosis is a rare and severe complication of oral anticoagulant therapy. Most commonly, it occurs in the first 10 days, and the incidence peaks between the third and sixth day of starting treatment. Cutaneous necrosis due to AC therapy is underreported in the literature, and studies refer to this condition as “coumarin-induced skin necrosis”; however, this term is not totally accurate, as coumarin itself has no anticoagulant properties. We report a case of a 78-year-old female patient with AC-induced skin necrosis, who presented with cutaneous ecchymosis purpura over her face, arms, and lower extremities 3 hours after AC intake.

## Introduction

Anticoagulants such as vitamin K antagonists (VKAs) are commonly used to treat thromboembolism [1]. Most oral anticoagulants come in the form of coumarin derivatives, which are taken by about 1-2% of adults worldwide as warfarin (WA) or acenocoumarol (AC). The most common side effect of these drugs is bleeding due to excessive lowering of the procoagulant factors [1,2]. AC is widely prescribed for prophylaxis of thromboembolic disorders. There are several advantages of AC over WA, such as shorter half-life, faster onset of action, and lack of reliance on CYP2C9. Chemically, it is (RS) 4-hydroxy-3-[1-(4-nitrophenyl)-3-oxobutyl] chromen-2-one [2]. Necrosis of the skin is a serious and infrequent complication of oral anticoagulant therapy. The most common time for this to occur is within the first 10 days of starting therapy. However, recent reports have described a late-onset presentation. Despite the lack of understanding of the pathogenesis of cutaneous necrosis, recent research has suggested that local and genetic factors, as well as a transient imbalance of coagulation mechanisms could play a role [3]. The most commonly used anticoagulant is WA, and most reports on anticoagulant-induced skin necrosis mention WA [4]. Cutaneous necrosis due to AC therapy is underreported in the literature. We report a case of a 78-year-old female patient with AC-induced skin necrosis, who presented with cutaneous ecchymosis purpura over her face, arms, and lower extremities 3 hours after AC intake.

## Case presentation

**Figure 1:**
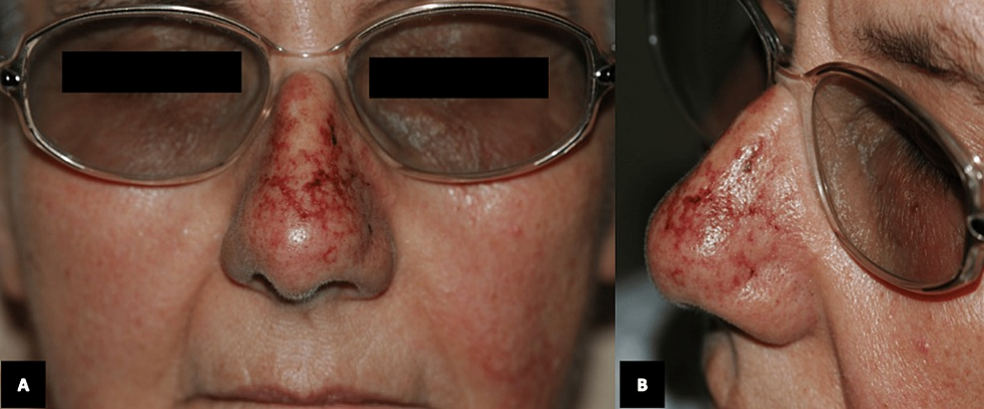
(A) From the front). (B) From the left side. Multiple ecchymotic and petechial spots can be noted over the face.

**Figure 2:**
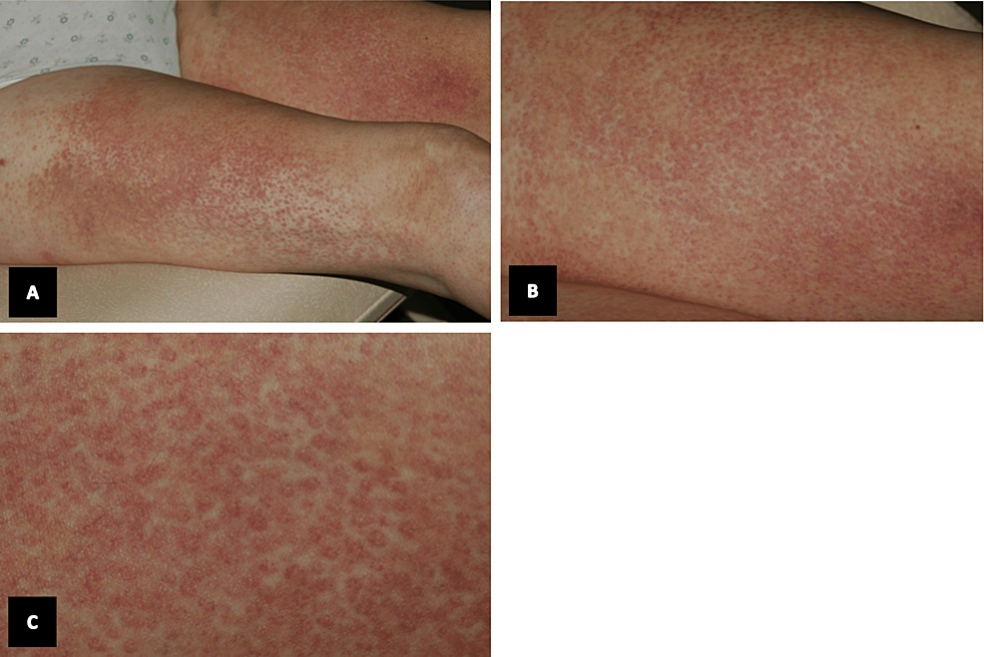
(A) Right and left thighs. (B and C) Zoomed picture of the right thigh. Multiple ecchymosis and purpuric spots can be noted over the lower extremities.

A 78-year-old female with a history of atrial fibrillation and hypercholesterolemia was brought to the emergency department with ecchymotic purpura over her face, especially the nose, arms, and lower extremities (Figures 1, 2), which started 3 hours after taking a dose of AC 4 mg by mouth (PO) once prior to admission. The patient denied any previous similar manifestation and no history of protein C or S deficiency in the family. Upon admission, current medications included aspirin 250 mg PO once daily, risedronate 5 mg PO once daily, amiodarone 200 mg PO once daily, and atorvastatin 10 mg PO at bedtime. Pertinent laboratory tests and monitoring upon admission included elevated fibrinogen at 5.4 g/L, sedimentation rate was 33 mm, C-reactive protein was 35 mg/L, gamma glutamyl transferase was 215 UI/L, and alkaline phosphatase was 207 UI/L. CBC and coagulation were normal, and protein C was 127 IU dL also in the normal range. Skin biopsy was taken from the right upper thigh. Histology showed a picture of purpura with thrombotic vasculitis. Photomicrograph showed intense blood extravasation with fibrin thrombi and necrosis of the small vessel wall surrounded by polymorphonuclear leukocytes, suggesting leukocytoclastic vasculitis and epidermal cleavage. A diagnosis of cutaneous necrosis provoked by AC was made. The patient was treated with vitamin K intravenously and fresh frozen plasma (FFP), after which her symptoms improved.

## Discussion

Despite the widespread use of oral anticoagulant treatment, reports of skin necrosis are still unusual. Recent theories support the combined roles of local factors, a temporary unbalance of coagulation mechanisms, and genetic factor that determines a decreased level of (protein C and protein S), the two vitamin-K dependent glycoproteins that lead to a hypercoagulable state in the pathogenesis of cutaneous necrosis induced by oral anticoagulants [3,5]. Skin necrosis can result from endothelial damage and microthrombi development. While coumarin does not have any anticoagulant effects by itself, it is the parent substance of congeners such as fluindione, AC (nicoumalone), bishydroxycoumarin (dicumarol), and phenprocoumon, which cause skin necrosis [4]. As a result, we will use the terminology used in prior studies [4] and refer to this disease as skin necrosis caused by coumarin congeners (SNICC).

The most often used of these is WA, and therefore it is mentioned in the majority of papers on anticoagulant-induced skin necrosis. Almost 0.01-0.1% of people on VKAs are thought to experience WA-induced skin necrosis. The first 10 days after initiating the medication are when SNICC most frequently happens [6]. During the third and sixth days, the incidence reaches its peak [7]. The process often begins in the first 10 days with a feeling of discomfort or pressure in the afflicted location, and then skin lesions suddenly arise. Later, symmetric purpuric lesions occur, group together to form distinct patches, develop hemorrhagic bullae, and eventually proceed to profound necrosis. However, several case reports have detailed a late-onset presentation, which can take months or years to manifest [7,8]. In our case, the patient developed her symptoms 3 hours after AC intake. SNICC has been reported most often in obese females over 50 years of age who have received thrombolytic therapy [7]. SNICC usually affects the areas with abundant subcutaneous fat such as the abdomen, buttocks, thighs, legs, and breasts in women, and the genitalia in men, but, rarely affects the face [9]. Little is mentioned regarding cutaneous necrosis induced by AC. We only found eight cases of AC-induced skin necrosis and leukocytoclastic vasculitis [5,9-17]. Summary of the clinical data is given in Table 1.

**Table 1:** Summary of the clinical data on acenocoumarol-induced skin necrosis and leukocytoclastic vasculitis F, female; NA, not applicable

Study	Sex/age	Time of onset	Location	Cutaneous manifestations	Histological findings
Cameron et al. (1974) [12]	F/70	3 years	Breast, thigh	Cutaneous necrosis	NA
Hofmann et al. (1982) [13]	F/36	Day 15	Breast, thigh	Cutaneous necrosis	NA
Susano et al. (1993) [14]	F/74	3 weeks	Lower limb	Palpable purpura	Leukocytoclastic vasculitis
Jiménez-Gonzalo et al. (1999) [15]	F/54	3 weeks	Lower limb	Erythema and purpura	Small vessel wall fibrinoid necrosis with surrounding primed neutrophils
Borrás-Blasco et al. (2004) [16]	F/76	2 months	Legs, abdomen, and arms	Purpura with vesicles	Leukocytoclastic vasculitis with IgA and complement deposition
Valdivielso (2004) [5]	F/82	Day 10	Lower limb	Cutaneous necrosis	Two samples were obtained: From the necrotic region (intense blood extravasation with fibrin thrombi and epidermal cleavage) and from the satellite purpuric lesion (leukocytoclastic vasculitis)
Aouam K et al. (2007) [17]	F/62	3 days	Lower limb	Purpuric lesions	Fibrinoid necrosis of the small vessel wall with surrounding primed neutrophils
Subramanian G et al. (2020) [9]	F/28	2 days	Right ear lobule	Edema, ecchymosis, and ulcer	NA
Our case	F/78	3 hours	Face, especially the nose, arms, and lower extremities	Ecchymotic purpura	Blood extravasation with fibrin thrombi and necrosis and leukocytoclastic vasculitis with epidermal cleavage

## Conclusions

Many distinct cutaneous adverse effects can result from oral anticoagulant therapy; however, SNICC is a rather uncommon adverse reaction. The most popular of them is WA, and therefore it is mentioned in the majority of papers on anticoagulant-induced skin necrosis. Most typically, this side effect appears 10 days after the commencement of treatment. Nonetheless, cases with a delayed onset have been documented. Typically, the belly, buttocks, thighs, legs, and breasts are affected by SNICC because of the abundance of subcutaneous fat in these locations. In the literature, cutaneous necrosis due to AC treatment is underreported.
